# The incidence and risk factors of asymptomatic primary spontaneous pneumothorax detected during health check-ups

**DOI:** 10.1186/s12890-017-0538-8

**Published:** 2017-12-07

**Authors:** Akihisa Mitani, Yukichika Hakamata, Megumi Hosoi, Masafumi Horie, Yoko Murano, Akira Saito, Shintaro Yanagimoto, Shoji Tsuji, Kazuhiko Yamamoto, Takahide Nagase

**Affiliations:** 1 Department of Respiratory Medicine, The University of Tokyo Hospital, 7-3-1, Hongo, Bunkyo-ku, Tokyo, 113-8655 Japan; 2 Division of Health Service Promotion, The University of Tokyo, 7-3-1, Hongo, Bunkyo-ku, Tokyo, 113-0033 Japan

**Keywords:** Primary spontaneous pneumothorax, Asymptomatic primary spontaneous pneumothorax, Medical check-ups, Chest X-rays, University students, Risk factors, Body height growth

## Abstract

**Background:**

Patients with primary spontaneous pneumothorax (PSP) usually complain of sudden-onset dyspnea and pleuritic chest pain. However, asymptomatic PSP has been incidentally detected on chest X-rays. In this study, we analyzed the incidence, characteristics, risk factors, and prognosis of asymptomatic PSP detected during regular medical check-ups in university students.

**Methods:**

In this study, 101,709 chest X-rays were performed during medical check-ups for students at the University of Tokyo between April 2011 and March 2016. Among them, 43 cases of asymptomatic PSP (0.042%) were detected. We calculated the lung collapse rate of pneumothorax using Kircher’s method. We also analyzed risk factors associated with asymptomatic PSP using characteristics inspected in medical check-ups.

**Results:**

The incidence of asymptomatic PSP was significantly higher in men than in women (0.050% vs 0.018%). Multivariate analysis revealed an association of younger age, greater height, lower body mass index, and greater height growth per year with an increased risk of asymptomatic PSP in male students. Mild lung collapse (<10%) was present in 22 of 43 students with asymptomatic PSP; among these, eight students eventually underwent an invasive therapy.

**Conclusions:**

The prevalence of asymptomatic PSP among university students was as high as 0.042%. In addition to known risk factors for conventional PSP, greater height growth was a risk factor for asymptomatic PSP. Careful follow-up is very important because a considerable number of patients with mild lung collapse eventually require an invasive medical procedure.

**Electronic supplementary material:**

The online version of this article (10.1186/s12890-017-0538-8) contains supplementary material, which is available to authorized users.

## Background

Pneumothorax is an abnormal collection of air in the pleural cavity resulting in lung collapse. Primary spontaneous pneumothorax (PSP) is not associated with any underlying lung disease and mainly occurs in young people. The incidence of PSP is 7.4–18 per 100,000 per year in males and 1.2–6 per 100,000 per year in females [1, 2].

Patients generally present with a sudden onset of chest pain with or without breathlessness. However, asymptomatic PSP is sometimes found on chest X-rays. Because PSP in some cases resolves spontaneously, asymptomatic PSP may develop and resolve in healthy individuals without being detected. In this study, we analyzed the incidence, characteristics, and risk factors of asymptomatic PSP detected during regular medical check-ups in university students.

## Methods

We examined chest X-rays performed during medical check-ups for students at the University of Tokyo between April 2011 and March 2016. Two physicians interpreted the radiographs independently, and pulmonologists confirmed the reports. We calculated the lung collapse rate of pneumothorax using Kircher’s method [3]. Rectangles were drawn from reference points to demarcate the outlines of the hemithorax and collapsed lung, and a percentage of pneumothorax is derived by subtracting the respective areas. Pneumothorax was classified into three groups using thresholds of 10% and 20% lung collapse.

We also analyzed characteristics inspected during medical check-ups, including age, body height, body weight, blood pressure, biochemical and urine tests, and electrocardiogram and respiratory symptoms. Height growth per year was calculated using body height from the present and the previous years. Students with pneumothorax who complained of any symptoms during the checkup were not included in the asymptomatic PSP group, although those who retrospectively recalled symptoms before the checkup were included.

This study was approved by the Institutional Review Board of the University of Tokyo (the approval number 14–71).

## Statistics

We used the chi-square test to compare students’ characteristics between the two groups and the Z-test to compare the asymptomatic PSP group to the whole student group. Multivariable logistic regression analysis was used to assess the association between students’ characteristics and asymptomatic PSP using a generalized estimation equation. The threshold for significance was a *p* value of <0.05 or an absolute z value of >1.96.

We performed all statistical analyses using SPSS version 22.0 (IBM SPSS Inc., Armonk, NY, USA).

## Results

A total of 101,709 chest X-rays were performed (78,678 males and 22,851 females), and 43 cases (0.042%) of asymptomatic PSP were detected (39 males and 4 females). None of them had underlying lung disease. The incidence of asymptomatic PSP in male students was 0.050%, which was significantly higher than that in female students (0.018%) (Table 1).

**Table 1:** Incidence of asymptomatic PSP (APSP) among the university students

	All case	(%)	APSP	(%)	*p*-value
Total students	101,529	(100.0%)	43	(0.042%)	
Sex					
Male	78,678	(77.5%)	39	(0.050%)	0.043*
Female	22,851	(22.5%)	4	(0.018%)	

*APSP*: asymptomatic primary spontaneous pneumothorax

*: *p* < 0.05

Table 2 shows the characteristics of students. Among male students, those with asymptomatic PSP were significantly younger compared with the average age of all male students. The average body height was significantly higher, and the average values of body weight and body mass index (BMI) were significantly lower in male students with asymptomatic PSP. We calculated height growth per year using body height from the present and the previous years, and the average height growth in the asymptomatic PSP group was significantly greater than that in all male students (0.62 ± 0.11 cm/year vs 0.21 ± 0.00 cm/year). There was no significant difference in systolic or diastolic blood pressure, but the average pulse rate was significantly higher in male students with asymptomatic PSP compared with that of all male students. As for female students, students with asymptomatic PSP had significantly greater height growth per year compared with the average height growth of all female students (1.13 ± 0.20 cm/year vs 0.17 ± 0.01 cm/year).

**Table 2:** Characteristics of students with asymptomatic PSP (APSP)

	All case			APSP				
	*N*	Mean	SEM	*N*	Mean	SEM	z-value	
Male								
Age	78,678	22.11	0.01	39	20.23	0.69	−3.20	*
BH (cm)	78,081	172.19	0.02	39	174.05	0.98	1.98	*
BW (kg)	78,081	62.95	0.03	39	56.58	1.05	−4.12	*
BMI	78,081	21.21	0.01	39	18.66	0.29	−5.46	*
BH change (cm/year)	43,769	0.21	0.00	34	0.62	0.11	3.59	*
sBP (mmHg)	78,646	119.93	0.05	39	116.26	1.83	−1.71	
dBP (mmHg)	78,646	66.42	0.03	39	66.03	1.31	−0.28	
PR (/min)	76,060	74.35	0.05	39	79.36	2.59	2.30	*
Female								
Age	22,851	23.04	0.03	4	24.75	4.21	0.74	
BH (cm)	22,642	159.69	0.04	4	164.40	0.51	1.72	
BW (kg)	22,641	51.03	0.05	4	50.80	2.84	−0.07	
BMI	22,641	19.99	0.02	4	18.80	0.93	−1.01	
BH change (cm/year)	12,003	0.17	0.01	3	1.13	0.20	2.75	*
sBP (mmHg)	22,837	107.11	0.08	4	101.75	4.23	−0.91	
dBP (mmHg)	22,836	61.93	0.05	4	59.25	5.41	−0.67	
PR (/min)	22,244	77.74	0.08	4	78.00	6.18	0.04	

*APSP* asymptomatic primary spontaneous pneumothorax, *BH* body height, *BW* body weight, *BMI* body mass index, *sBP* systolic blood pressure, *dBP* diastolic blood pressure, *PR* pulse rate

*: |z| > 1.96

During the 5 years between the ages of 18 and 23 years, mean body height increased from 171.4 ± 0.1 cm to 172.5 ± 0.1 cm in male students and from 158.8 ± 0.1 cm to 160.0 ± 0.1 cm in female students (Additional file 1: Figure S1). During the 5 years between the ages of 23 and 28 years, there was little to no change in mean body height. However, there was only a low correlation between individual height growth and age (*r* = −0.2375). Therefore, we included both age and height growth as independent variables in the multivariate analysis.

There were no differences in biochemical test results between the asymptomatic PSP group and all students (Additional file 2: Table S1). As for urinary tests, only one student with asymptomatic PSP had proteinuria, and none of the students with asymptomatic PSP had abnormal echocardiogram findings (data not shown).

Results of the multivariable logistic regression analysis of factors associated with asymptomatic PSP in male students are listed in Table 3. Asymptomatic PSP was associated with younger age, greater body height, and lower BMI. It was also associated with greater body height growth (odds ratio, 1.864; 95% confidence interval, 1.248–2.783; *p* = 0.002). We did not perform the analysis in female students because of the small number of asymptomatic PSP cases.

**Table 3:** Multivariable logistic regression analysis for asymptomatic PSP

Male					
	Odds Ratio	95% CI		*p*-value	
age	0.758	0.603	0.953	0.018	*
BH	1.065	1.002	1.132	0.042	*
BMI	0.609	0.511	0.725	<0.001	***
BH change	1.864	1.248	2.783	0.002	**

*BH* body height, *BW* body weight, *BMI* body mass index

*: *p* < 0.05, **: *p* < 0.01, ***: *p* < 0.001

We calculated the lung collapse rate in pneumothorax and classified pneumothorax into three groups using thresholds of 10% and 20% lung collapse. The mild group (22 cases) showed less than 10% lung collapse, the moderate group (12 cases) showed 10% to 20% lung collapse, and the severe group (9 cases) showed lung collapse of 20% or more.

**Fig. 1:**
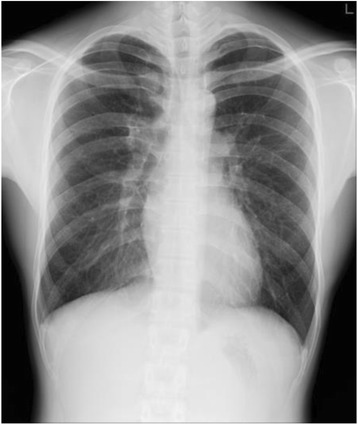
Case 1: A 20-year-old man. Left pneumothorax: lung collapse rate 3.9%

The following are representative cases. Case 1: a 20-year-old male student underwent a chest X-ray during a medical checkup and left pneumothorax (lung collapse rate 3.9%) was detected (Fig. 1). We informed the patient and he consulted with a clinic 9 days later, and a follow-up chest X-ray confirmed that pneumothorax had already resolved. He had no respiratory symptoms and was a never-smoker. He had a history of previous left PSP.

**Fig. 2:**
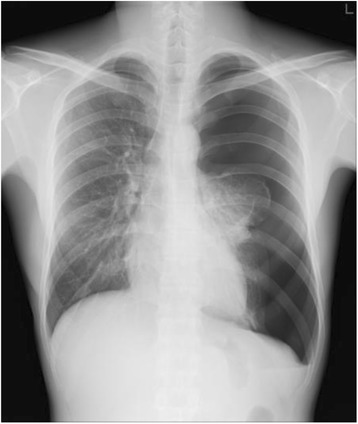
Case 2: A 24-year-old man. Left pneumothorax: lung collapse rate 66.7%

Case 2: a 24-year-old man underwent a medical checkup without any symptoms. Left pneumothorax (lung collapse rate 66.7%) was detected on a chest X-ray (Fig. 2). We called the patient the same day; he was hospitalized and underwent chest drainage and surgical treatment. He was a never-smoker and had no history of previous PSP.

**Fig. 3:**
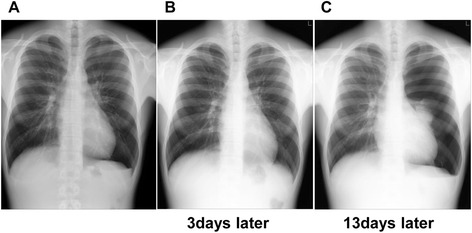
Case 3: A 19-year-old man. **a** Left pneumothorax: lung collapse rate 5.7%. **b** 3 days later. Improved spontaneously. **c** 13 days later. Relapsed: lung collapse rate 63.3%

Case 3: a 19-year-old male student had a chest X-ray in a medical checkup without any respiratory symptoms. Left pneumothorax (lung collapse rate 5.7%) was detected (Fig. 3a). We informed the patient, he consulted with a clinic 3 days later, and it was confirmed that that pneumothorax had spontaneously healed (Fig. 3b). However, it relapsed 10 days later (Fig. 3c), and he was hospitalized and underwent surgical treatment. He was a never-smoker and had no history of previous PSP.

The characteristics of asymptomatic PSP are summarized in Table 4. Patients in the severe group were significantly more likely to have pleural effusion, but there was no difference in laterality or the existence of previous PSP. Among the 43 students, 5 recalled some symptoms after the detection of asymptomatic PSP. Four students had chest pain before the medical checkup, but it had disappeared by the checkup and they did not mention it. One student had no symptoms on the day of the checkup, but he developed a cough a few days later. Another few days passed until asymptomatic PSP was detected on his chest X-ray. There was no correlation between these symptoms and the severity of lung collapse. As for therapy, patients with severe pneumothorax did not have a significantly higher rate of invasive therapy. In contrast, more than one third of patients in the mild or moderate group eventually needed invasive therapy (Table 4, Additional file 3: Figure S2). Among these patients, there were two cases with recurrence within 2 weeks after spontaneous resolution.

**Table 4:** Comparison among different severity levels of asymptomatic PSP

		Total	Mild		Moderate		Severe			
			*n* = 22	(%)	*n* = 12	(%)	*n* = 9	(%)	*p*-value	
Right or left										
	right	23	9	(40.9%)	9	(75.0%)	5	(55.6%)	0.161	
	left	20	13	(59.1%)	3	(25.0%)	4	(44.4%)		
Pleural effusion										
	(−)	30	20	(90.9%)	9	(75.0%)	1	(11.1%)	<0.001	***
	(+)	13	2	(9.1%)	3	(25.0%)	8	(88.9%)		
Previous pneumothorax										
	(−)	32	15	(68.2%)	9	(75.0%)	8	(88.9%)	0.486	
	(+)	11	7	(31.8%)	3	(25.0%)	1	(11.1%)		
Recalled symptom										
	(−)	38	20	(90.9%)	10	(83.3%)	8	(88.9%)	0.804	
	(+)	5	2	(9.1%)	2	(16.7%)	1	(11.1%)		
Therapy										
	conservative	18	11	(50.0%)	5	(41.7%)	2	(22.2%)	0.452	
	invasive	18	8	(36.4%)	4	(33.3%)	6	(66.7%)		
	N/A	7	3	(13.6%)	3	(25.0%)	1	(11.1%)		

***: *p* < 0.001

## Discussion

Medical check-ups revealed 43 cases (0.042%) of asymptomatic PSP among 101,709 chest X-rays. The incidence is comparable to the annual incidence of conventional PSP in men (0.007%–0.018%) [1, 2], suggesting that asymptomatic PSP may develop in a healthy population more often than previously believed. The predominance of males presenting with asymptomatic PSP is consistent with conventional PSP [4, 5]. The incidence of PSP detected during medical check-ups was reported to be 0.003%–0.033% in the previous studies on a relatively small number of chest X-rays [6, 7]. The relatively high incidence in our study is partly explained by the fact that the medical check-ups were performed on university students, who are a younger population. Another explanation may be the clustering of PSP because of changes in atmospheric pressure [8] or levels of air pollution [9]. However, 5 year research period should minimize the impact of these effects.

Characteristics of asymptomatic PSP are similar to those of conventional PSP [4, 5]. Male students with asymptomatic PSP were likely to be younger, taller, and thinner compared with the average measurements for all male students. More interestingly, their height growth per year was likely to be greater. Multivariable logistic regression analysis also revealed that asymptomatic PSP was associated with younger age, greater body height, lower BMI, and greater body height growth. Among these, greater height growth has not previously been reported as a risk factor for PSP. It is possible that rapid height growth increases the gradient of negative pleural pressure, but lungs are still immature and easily develop subpleural blebs. Furthermore, although they had no symptoms, male students with asymptomatic PSP showed increased pulse rates. Rapid pulse rates could be the only sign of asymptomatic PSP. However, the difference is too small for differential diagnosis. The small number of cases in female students made statistical analysis difficult, but female students with asymptomatic PSP also showed greater body height growth.

There is reportedly a poor correlation between pain intensity and pneumothorax size [10, 11]. Therefore, it was not surprising that 9 of 43 (20.9%) asymptomatic PSP cases showed lung collapse of 20% or more. But it should be emphasized that there was no significant correlation between the prognosis and the severity of asymptomatic PSP, although patients with severe PSP tended to undergo invasive treatment. O’Rourke and Yee reported that up to 80% of pneumothorax cases with collapse of less than 15% volume of the lung will have no persistent air leak and recurrence when managed with observation alone [12]. In our study, however, more than one third of patients with mild or moderate lung collapse eventually underwent the invasive therapy. Furthermore, some patients had a recurrence within 2 weeks of a recovery. Careful follow-up is very important because observation alone may not be sufficient for the treatment of asymptomatic PSP.

Several limitations of this study should be acknowledged. First, tobacco smoking, which is an important risk factor for PSP [2], was not included in this analysis because we do not have the smoking rate for university students. However, among the 43 students with asymptomatic PSP, only one was a current smoker and one was an ex-smoker. The smoking rate of students with asymptomatic PSP was not high compared with that of males in their twenties in Japan (about 30%) [13]. Second, most cases of asymptomatic PSP, especially mild or moderate cases, were detected a few days after the medical checkup, and the follow-up observation was sometimes insufficient. Immediate radiographic image interpretation is desirable.

## Conclusions

The prevalence of asymptomatic PSP among university students was not negligible (0.042%). Younger age, greater body height, lower BMI, and greater height growth were risk factors for asymptomatic PSP. Because the severity of asymptomatic PSP was not correlated with the prognosis, careful follow-up observation is essential.

## Additional files

Additional file 1: Figure S1. The average body height at each age. During the 5 years between the ages of 18 and 23 years, mean body height increased both in male and female students. During the next 5 years, however, there was little to no change in mean body height. (PPT 301 kb)

Additional file 2: Table S1. Biochemical examinations of students with asymptomatic PSP (APSP). There were no differences in biochemical test results between the asymptomatic PSP group and all students. (DOCX 16 kb)

Additional file 3: Figure S2. The lung collapse rate and therapy in each case. The severity of asymptomatic PSP was not correlated with the prognosis. (PPT 255 kb)

## Abbreviations

BMI: Body mass index
PSP: Primary spontaneous pneumothorax
