# Supplementary figures and images for: The incidence and risk factors of asymptomatic primary spontaneous pneumothorax detected during health check-ups
Source: BMC Pulm Med. 2017 Dec 7;17:177. doi: 10.1186/s12890-017-0538-8 (PMC5721680; doi:10.1186/s12890-017-0538-8)

## Slide 1
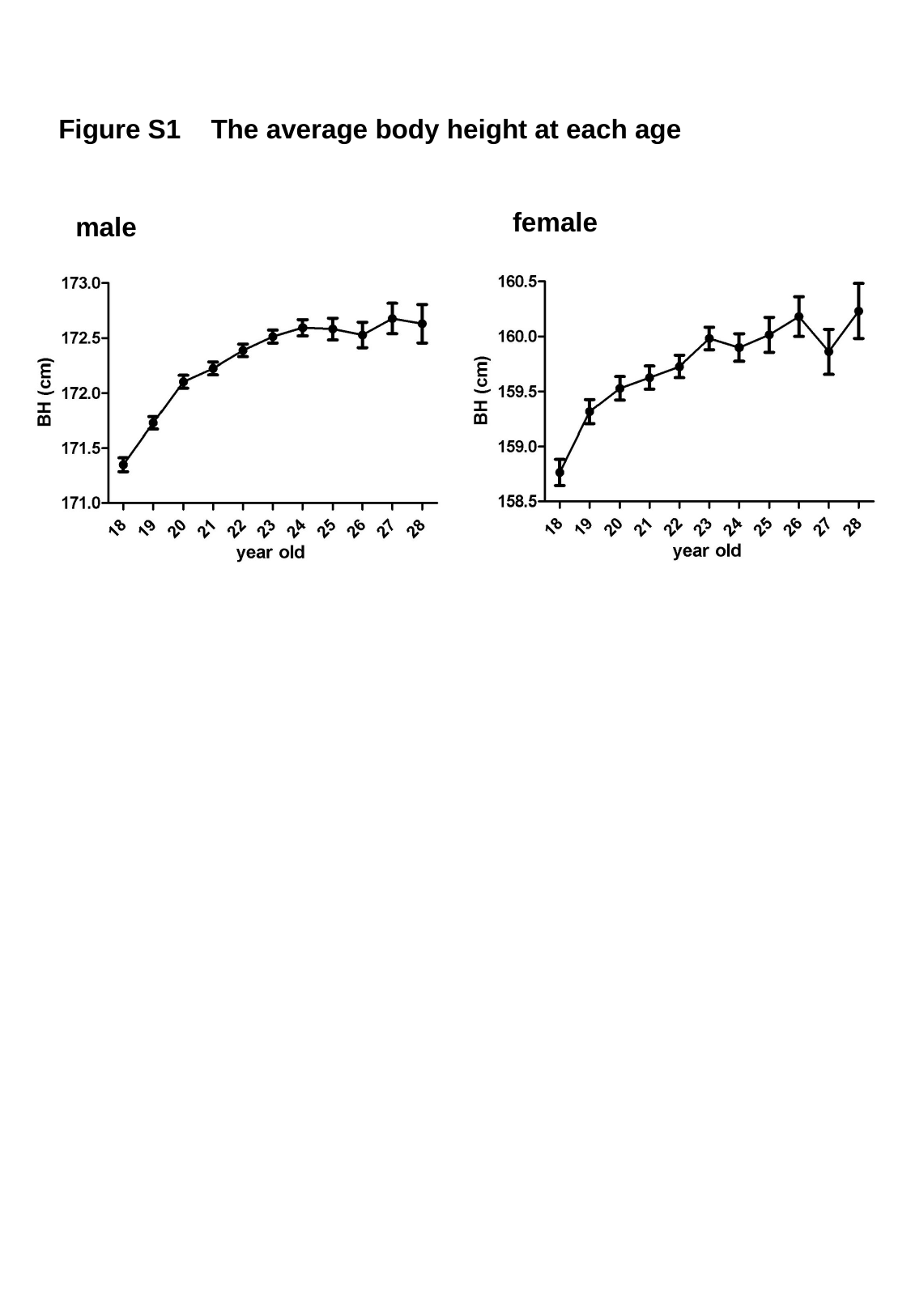

Figure S1 The average body height at each age
female
male

Supplement: Supplementary file 1 — The average body height at each age. During the 5 years between the ages of 18 and 23 years, mean body height increased both in male and female students. During the next 5 years, however, there was little to no change in mean body height. (PPT 301 kb) [file 12890_2017_538_MOESM1_ESM.ppt]

## Slide 1
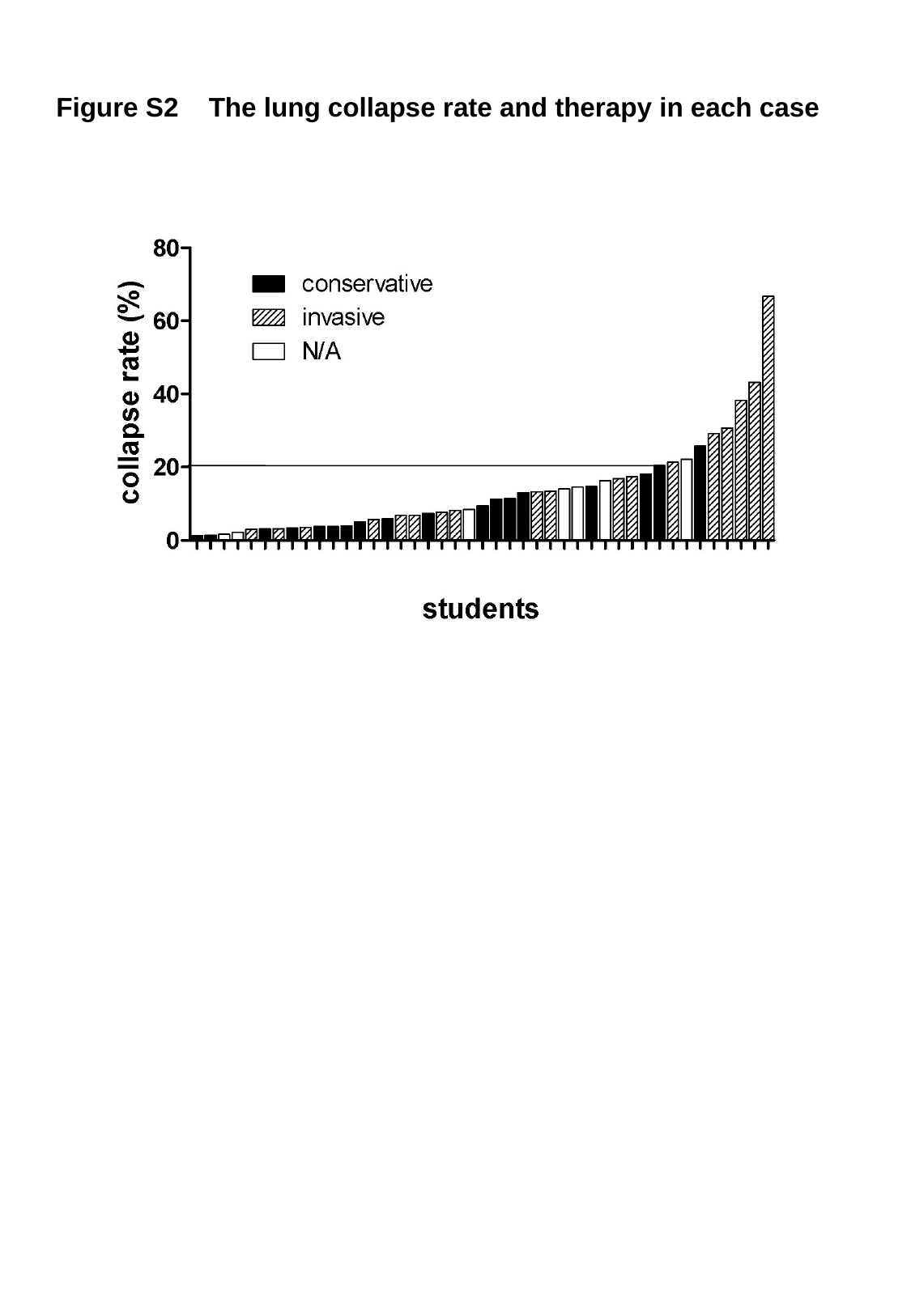

Figure S2 The lung collapse rate and therapy in each case

Supplement: Supplementary file 3 — The lung collapse rate and therapy in each case. The severity of asymptomatic PSP was not correlated with the prognosis. (PPT 255 kb) [file 12890_2017_538_MOESM3_ESM.ppt]
