# Supplementary material for: The incidence and risk factors of asymptomatic primary spontaneous pneumothorax detected during health check-ups
Source: BMC Pulm Med. 2017 Dec 7;17:177. doi: 10.1186/s12890-017-0538-8 (PMC5721680; doi:10.1186/s12890-017-0538-8)
Supplement: Supplementary file 2 — Biochemical examinations of students with asymptomatic PSP (APSP). There were no differences in biochemical test results between the asymptomatic PSP group and all students. (DOCX 16 kb) [file 12890_2017_538_MOESM2_ESM.docx]

Table S1 Biochemical examinations of students with asymptomatic PSP (APSP)

| **male** |  |  |  |  |  |  |  |  |
| --- | --- | --- | --- | --- | --- | --- | --- | --- |
|  | **all case** |  |  | **APSP** |  |  |  |  |
|  | **N** | **Mean** | **SEM** | **N** | **Mean** | **SEM** | **z-value** |  |
| **GOT (IU/l)** | **28,162** | **21.47** | **0.09** | **25** | **22.00** | **1.48** | **0.18** |  |
| **GPT (IU/l)** | **22,446** | **21.53** | **0.13** | **25** | **19.32** | **2.30** | **-0.57** |  |
| **LDL (mg/dl)** | **28,161** | **93.93** | **0.15** | **25** | **88.08** | **5.31** | **-1.17** |  |

| **female** |  |  |  |  |  |  |  |  |
| --- | --- | --- | --- | --- | --- | --- | --- | --- |
|  | **all case** |  |  | **APSP** |  |  |  |  |
|  | **N** | **Mean** | **SEM** | **N** | **Mean** | **SEM** | **z-value** |  |
| **GOT (IU/l)** | **8,515** | **18.00** | **0.07** | **2** | **22.00** | **1.00** | **0.87** |  |
| **GPT (IU/l)** | **6,760** | **13.19** | **0.12** | **2** | **25.00** | **11.00** | **1.73** |  |
| **LDL (mg/dl)** | **8,516** | **93.78** | **0.25** | **2** | **78.00** | **10.00** | **-0.95** |  |

APSP: asymptomatic primary spontaneous pneumothorax.
